# Pathogenesis and treatment of neuropsychiatric systemic lupus erythematosus: A review

**DOI:** 10.3389/fcell.2022.998328

**Published:** 2022-09-05

**Authors:** Yuhong Liu, Zhihua Tu, Xi Zhang, Keqian Du, Zhengquan Xie, Zhiming Lin

**Affiliations:** ^1^ Department of Rheumatology, Third Affifiliated Hospital of Sun Yat-sen University, Guangzhou, China; ^2^ Department of Rheumatology, Panyu Hospital of Chinese Medicine, Guangzhou, China

**Keywords:** SLE, NPSLE, pathogenesis, treatment, autoimmune diseases

## Abstract

Systemic lupus erythematosus (SLE) is an autoimmune inflammatory disease with a complex pathogenesis. Neuropsychiatric systemic lupus erythematosus (NPSLE) is a serious complication of SLE that involves the nervous system and produces neurological or psychiatric symptoms. After decades of research, it is now believed that the diverse clinical manifestations of NPSLE are associated with intricate mechanisms, and that genetic factors, blood-brain barrier dysfunction, vascular lesions, multiple autoimmune antibodies, cytokines, and neuronal cell death may all contribute to the development of NPSLE. The complexity and diversity of NPSLE manifestations and the clinical overlap with other related neurological or psychiatric disorders make its accurate diagnosis difficult and time-consuming. Therefore, in this review, we describe the known pathogenesis and potential causative factors of NPSLE and briefly outline its treatment that may help in the diagnosis and treatment of NPSLE.

## 1 Introduction

Systemic lupus erythematosus (SLE) is an autoimmune inflammatory disease of unknown origin, and its pathogenesis involves genetic factors, epigenetics, and environmental factors that lead to abnormal immune function (Tsokos et al., 2016). Neuropsychiatric systemic lupus erythematosus (NPSLE) is a devastating complication of SLE that involves the nervous system and produces neurological or psychiatric symptoms with a poor prognosis and high mortality. NPSLE involves both the central and peripheral nervous system, and its symptoms can range from subtle abnormalities to significant manifestations such as headache, cerebrovascular lesions, cognitive dysfunction, epilepsy and acute disorders of consciousness (Bertsias & Boumpas, 2010). According to the American College of Rheumatology (ACR) nomenclature and classification criteria, NPSLE includes 12 neuropsychiatric symptoms related to the central nervous system and seven related to the peripheral nervous system (Table 1), in addition to neurological syndromes of the autonomic nervous system The American College, 1999). In this review, we concentrate on the pathogenesis of neuropsychiatric systemic lupus erythematosus and describe its epidemiology and treatment accordingly.

**TABLE 1:** Clinical symptoms in NPSLE.

	Focal	Diffuse
Central Nervous System	Headache	Psychosis Mood disorders Anxiety disorders Cognitive dysfunction Acute confusional state
	Myelopathy	Mood disorders
	Seizure disorder	Anxiety disorders
	Movement disorders	Cognitive dysfunction
	Cerebrovascular disease	Acute confusional state
	Aseptic meningitis	—
	Demyelinating syndromes	—
Peripheral Nervous System	Acute inflammatory demyelinating lesions (Grimm-Barré syndrome)	—
	Autonomic neuropathy	
	Myasthenia gravis	
	Polyneuropathy	
	Mononeuropathy	
	Cranial neuropathy	
	Plexopathy	

## 2 Manuscript formatting

### 2.1 Prevalence and epidemiology

Due to the heterogeneity of different studies, the reported prevalence of NPSLE ranged from 12 to 95%, which is mainly related to the sample size and exclusion criteria of each study, the definition of different cases, and also influenced by different races and regions. In a meta-analysis by Unterman et al. which pooled all available studies, the prevalence of NPSLE was 44.5% in prospective studies but only 17.6% in retrospective studies (Unterman et al., 2011).

NPSLE is more frequently reported in African and Asian descendants compared to Caucasian people, whereas white patients with NPSLE had higher disease activity and more severe manifestations (Sarwar et al., 2021). A 3-years prospective study that followed 370 SLE patients with no previous history of central nervous system (CNS) involvement concluded that the incidence of CNS involvement was only 4.3% and the prevalence was 7.8/100 person years (Kampylafka et al., 2013). Another study analyzed 308 patients with a confirmed diagnosis of SLE in China. The estimated incidence rate of NPSLE was 12.4% (Zhang et al., 2020). A single center study noted that the prevalence of NPSLE among Chinese patients with SLE was only 6.4%, which is mainly because there are no exact diagnostic criteria. On the one hand, mild manifestations of NPSLE may not be included in the diagnosis, and on the other hand, not all psychiatric symptoms are associated with NPSLE (Li et al., 2020).

A recent systematic review and results from the Swiss lupus cohort study pointed out that the average prevalence of NPSLE ranged from 10.6 to 96.4%. In the Swiss SLE cohort study, the prevalence of NPSLE was 28.1% and cerebrovascular insults, seizures and psychosis occurred in 7.1, 5.3 and 6.5% respectively (Meier et al., 2021). Vivaldo et al. analyzed the different criteria of NPSLE published to date and assessed their advantages and limitations. They noted that, while the 1999 ACR criteria made significant progress in unifying the criteria for NPSLE, its reported incidence still varies widely due to the lack of a completely established pathophysiology. Therefore, more comprehensive tools are needed, and several models have been designed to improve the attribution of neuropsychiatric symptoms to SLE (Vivaldo et al., 2018).

### 2.2 Pathogenesis

The diverse clinical manifestations of NPSLE are associated with intricate mechanisms. The pathogenesis of NPSLE is multifactorial, involving a variety of inflammatory cytokines, genetic factor, multiple autoimmune antibodies, blood-brain barrier dysfunction, complement activation and immune complexes, which contribute to vasculopathy, cytotoxicity and autoantibody-mediated neuronal damage (Table 2). However, the pathogenic processes that lead to neurological damage and consequential pathophysiological changes and clinical manifestations in SLE patients are still largely unknown.

**TABLE 2:** Factors associated with the pathogenesis of NPSLE.

	—
Genetic	FcγRIIIa, FcγRIIIb genotypes
	ITGAM genotypes
Blood-brain barrier dysfunction and Cerebrovascular lesions	Albumin cerebrospinal fluid/serum ratio
	S100 calcium-binding protein B
	Microglia activation
	Abnormal endothelial-immune cell interactions
Autoantibodies	Antiphospholipid (aPL)
	Anti-ribosomal P protein
	Anti-N-Methyl-d-Aspartate (NMDA) receptor
	Anti-aquaporin 4
	Anti-endothelial
	Anti-ubiquitin carboxyl hydrolase L1(UCH-L1)
	Anti-glyceraldehyde 3-phosphate dehydrogenase (GAPDH)
Complement activation	Anti-C1q
	C3/AP50
	C4 and C5
Cytokines	Inflammatory factor: TNF-α, TWEAK, IFN-γ, IL-6, IL-8, IL-10, BAFF
	Chemokines: MCP-1/CCL2, IP-10/CXCL10, G-CSF, GM-CSF

#### 2.2.1 Genetic factor

Numerous studies have illustrated that genetic factors are closely associated with the development of SLE, and early literature reported that there is familial aggregation of SLE and that asymptomatic relatives often have circulating autoantibodies. However, at the same time, ethnic differences, genetic heterogeneity, low epistasis, and the role of environmental factors have made it difficult to investigate susceptibility genes for human SLE. In particular, the various genotypes involved in the pathogenesis of NPSLE remain uncertain. A meta-analysis of genetic variants associated with NPSLE signified that among the many genotypes with possible association with NPSLE, FcγRIIIa, FcγRIIIb and ITGAM genotypes are potential susceptibility genes for NPSLE (Ho et al., 2016). The identification of functional genetic susceptibility loci for NPSLE, the discovery of finely regulated pathogenic gene expression, and the elucidation of the molecular mechanisms involved may provide new ideas for early detection, diagnosis and treatment of NPSLE.

#### 2.2.2 Blood–brain barrier dysfunction

The blood-brain barrier (BBB) is an essential physiological barrier structure that can isolate the central nervous system (CNS) from the peripheral blood circulation and maintain the stability of the internal environment of the central nervous system by limiting toxic substances and inflammatory factors in the blood, selectively transporting nutrients needed by brain tissue, and excluding toxic substances and metabolites produced by brain tissue (Obermeier et al., 2013). Previous studies have generally held the view that the permeability of the BBB plays a major contributing role in the pathogenesis of NPSLE (Stock et al., 2017).

The presence of the albumin cerebrospinal fluid (CSF)/serum ratio is regarded as a surrogate marker of serum penetration into the CNS and can be used to assess the BBB permeability (Fanali et al., 2012; Wang et al., 2018). S100 calcium-binding protein B (S100 B) is of major relevance in cell proliferation, differentiation, gene expression, and apoptosis. Under physiological conditions, it is a neurotrophic factor that affects the growth, proliferation, and differentiation of glial cells, maintains calcium homeostasis, and promotes brain development. Under normal conditions, S100 B does not cross BBB. When the BBB is damaged, S100 B leaks out of the cytosol into the cerebrospinal fluid and then into the blood, resulting in an increase in the concentration of S100 B in the blood (Rothermundt et al., 2003; Michetti et al., 2012). In neurological disorders, S100 B has a prospective role as a neurological screening tool and biomarker of central nervous system injury (Sen & Belli, 2007).

Microglia are intrinsic immune effector cells within the CNS and play an extremely critical role in the physiological processes of the CNS by mediating endogenous immune responses to CNS injury and disease. Nestor J et al. identified microglia activation in the DNRAb-mediated cognitive impairment in a mouse model of lupus-prone and concluded that inhibition of microglia activation can attenuate the manifestation of NPSLE in a variety of murine models (Nestor et al., 2018). In parallel, it has been previously demonstrated that inhibition of microglia activation can reduce synaptic loss and behavioral phenotypic changes in NPSLE model mice, suggesting that microglia play an important role in the physiological and pathological processes of NPSLE (Bialas et al., 2020).

Furthermore, the mechanism of the neurological dysfunction of SLE may be related to abnormal endothelial-immune cell interactions that lead to the entry of immune cells as well as various autoantibodies across the BBB into the CNS. The severity of BBB injury exerts a substantial contributing effect on the development of the acute confusional state as a result of the accelerated entry of large numbers of anti-N-methyl-d-aspartate receptor NR2 antibodies into the CNS (Hirohata et al., 2014). S. Gelb et al. investigated the function of brain barriers in the MRL/lpr mice and indicated that the abnormal disorder of the blood-cerebrospinal fluid barrier (BCSFB) is the basis of brain exposure to neuropathic autoantibodies. TWE**A**K/Fn14 interactions have a significant impact on the pathogenesis of NPSLE by boosting inflammatory cell buildup in the choroid plexus, compromising BBB integrity and enhancing neuronal damage (Gelb et al., 2018).

#### 2.2.3 Cerebrovascular lesions

At present, research on the cerebrovascular aspects of the pathogenesis of NPSLE is still relatively restricted. Autopsy findings of NPSLE patients suggest that their CNS damage is related to cerebrovascular lesions (Jeltsch-David & Muller, 2014). Cerebral microvascular ischemia and thrombosis, small vessel non-inflammatory lesions, focal vascular occlusion, and microhemorrhage are common pathological manifestations. The process of thrombosis of the large and small intracranial vessels caused by leukocyte coagulation and accelerated atherosclerosis is also involved in part in the pathogenesis of NPSLE. Secondly, immune complex deposition, complement activation and multiple autoantibody-mediated vascular injury also make pivotal contributions to the etiology of NPSLE (Sarwar et al., 2021; Thirunavukkarasu et al., 2021). Further clarification of the specific mechanisms involved and their interrelationships may have the potential for more specific updates on the treatment of neuropsychiatric symptoms in NPSLE.

In addition to cerebrovascular involvement, brain histology of NPSLE patients also showed cerebral edema, vascular remodeling and wall calcification, neuronal and myelinated axonal loss, microinfarcts and diffuse ischemic changes, microglia proliferation and reactive astrocytosis, which also indicated that microglia activation may contribute to disease development by affecting neuronal and synaptic structure and function. These aforementioned pathological changes eventually lead to focal or diffuse brain edema or diffuse endothelial injury, which further leads to disruption of the blood-brain barrier (Sibbitt et al., 2010).

#### 2.2.4 Autoantibodies

An overriding feature of SLE is the production of autoantibodies, and several antibodies have been identified to be associated with NPSLE manifestations. Up to now, over 116 antibodies have been reported in SLE, of which at least 20, including 11 brain-specific and nine systemic antibodies, have been associated with NPSLE (Sciascia et al., 2014). However, available findings on the significance of different autoantibodies in NPSLE are controversial. The mechanism of the specific neuropsychiatric manifestations of SLE induced by any one autoantibody has not been elucidated.

##### 2.2.4.1 Antiphospholipid antibodies

Antiphospholipid (aPL) antibodies include lupus anticoagulant (LA), anticardiolipin (aCL) antibody, anti-β2 glycoprotein-I antibody (anti-β2GPI) and anti-phosphatidylserine antibody (aPS). They are involved in vascular endothelial cell injury, platelet activation and thrombosis, resulting in focal cerebral ischemia and intracranial vascular embolism. From previous studies on autoantibodies in NPSLE, it is not difficult to observe that aPL has been one of the most widely studied autoantibodies. Numerous studies have demonstrated the relationship between aPL antibodies and focal neurological manifestations of NPSLE, such as headache, stroke, and epilepsy, as well as diffuse neurological manifestations, including cognitive dysfunction.

The role of aPL antibodies in the course of SLE is now known to be as follows. Firstly, aPL antibodies activate endothelial cells, platelets and monocytes, which may lead to prothrombotic particles. Secondly, aPL antibodies can accelerate atherosclerosis, which is an independent risk factor for cerebrovascular ischaemia. In addition, aPL antibodies are associated with diffuse NPSLE syndromes such as seizures, chorea, cognitive dysfunction and myelopathy, suggesting that these autoantibodies have pathogenic effects beyond their pro-thrombotic role (Gris et al., 2015; Fleetwood et al., 2018).

S. Sciascia et al. concluded that aPL antibodies (in particular LA) were remarkably correlated with cerebrovascular events in NPSLE by analyzing the main characteristics of 19 articles on 6,239 patients with aPL antibodies and NPSLE (Sciascia et al., 2014). By employing the same electronic database search methodology, the findings of Donnellan C et al. illustrate that cognitive impairment is prevalent in aPL antibodies patients (including APS, SLE, and NPSLE), and there is a considerable degree of association with neuroimaging biomarkers. Unfortunately, aPL antibodies are not present in all patients with cognitive impairment, so the link between cognitive impairment and neuroimaging biomarkers reported in their study cannot be definitively established (Donnellan et al., 2021). In addition, Meier et al. conducted a systematic review of the existing literature and proved that the augment in the value of aPL antibodies is related to the existence of cerebrovascular disease (Meier et al., 2021).

Further clarification of the interrelationship existing between neuroimaging biomarkers and cognitive impairment in aPL antibodies positive patients may be advantageous in guiding future treatment and prognostic improvement in cognitive dysfunction in NPSLE.

##### 2.2.4.2 Anti-ribosomal P protein antibodies

Anti-ribosomal P protein antibodies, one of the specific autoantibodies for SLE, appear in up to 46% of SLE patients and are also thought to be associated with NPSLE, particularly diffuse psychiatric/neuropsychological syndromes (Eber et al., 2005; Abdel-Nasser et al., 2008). A previous meta-analysis indicated that the estimated sensitivity and specificity of anti-ribosomal P protein antibodies for NPSLE were 26 and 80%, respectively, which was reconfirmed by some later studies (Karassa et al., 2006; Abdel-Nasser et al., 2008). It was concluded that anti-P antibodies have limited diagnostic value and do not aid in the identification of disease phenotypes (such as psychosis, mood disorders, and other diffuse or focal manifestations). Matus et al. demonstrated that anti-P antibodies bind to a novel neuronal cell surface protein distributed in regions involved in memory, cognition, and emotion and trigger neurotoxic effects through the rapid influx of Ca2+ into neurons, leading to apoptosis (Matus et al., 2007).

The results of Bravo-Zehnder et al. suggest that anti-P antibodies induce apoptosis and dysfunction by interacting with hippocampal neurons that express the neuronal surface P antigen. When the BBB is disrupted, anti-P antibodies can directly enter the hippocampus to cause memory impairment in mice without neuronal death, which may be one of the potential mechanisms of cognitive/memory dysfunction in anti-P antibody-positive SLE patients (Bravo-Zehnder et al., 2015). Furthermore, the circulating anti-P antibodies attack the endothelial cells in the BBB, leading to endothelial cell stimulation to produce pathogenic cytokines and chemokines that further inflammation of the blood vessels and impairment of the BBB, which penetrates into the brain, resulting in inflammation of the brain (Yoshio et al., 2016). A recent prospective study by Arinuma Y et al. illustrated that the presence of anti-ribosomal P protein antibodies in serum significantly increases mortality in patients with diffuse NPSLE and is considered a remarkable risk factor for its poor prognosis. However, whether it is a specific prognostic factor has not been demonstrated (Arinuma et al., 2019).

Despite the fact that the mechanisms by which anti-P antibodies cause memory and cognitive impairment are not fully understood, these results are evidence of the potentially neuropathogenic nature of anti-P antibodies and also represent a molecular target for future exploration of neuropsychiatric disorders of NPSLE and other psychiatric diseases.

##### 2.2.4.3 Anti-N-methyl-d-aspartate receptor antibodies

The N-Methyl-d-Aspartate (NMDA) receptor is a subtype of the excitatory glutamate receptor, which is a key substance in learning and memory. Excessive activation of NMDA receptors can cause an ionic imbalance within and outside the cell membrane, activating neurotoxic signaling pathways and ultimately resulting in a range of neuronal dysfunctions and impaired cognitive function (Vyklicky et al., 2014). By establishing a mouse model of autoimmune encephalitis, Planagumà J et al. provided reliable evidence that anti-NMDAR antibodies can alter memory and behavior in mice through reducing cell surface and synaptic NMDAR. The results open up the possibility of using treatments that decrease the number of antibodies or antibody-producing cells (Planagumà et al., 2015).

Through a multivariate linear regression analysis of a cohort of SLE patients, Gono et al. discovered that NPSLE was the most conspicuous independent variable associated with anti-N-methyl-d-aspartate receptor subunit 2A (NR2A) antibody positivity. This suggests that serum anti-NR2A antibodies may be linked to NPSLE and its complications, and may accumulate in non-neural organs as well (Gono et al., 2011). A review of glutamate receptor antibodies in neurological disorders concluded that anti-NMDA-NR1 antibodies and anti-NMDA-NR2A/B antibodies are generally present in subgroups of individuals with SLE, NPSLE, Sjogren’s syndrome, epilepsy, encephalitis, cerebellar ataxia, schizophrenia, mania or stroke. These autoimmune anti-glutamate receptor antibodies activate glutamate receptors in neurons, causing a drastic reduction in the expression of membrane-like NMDA receptors in neurons, impairing glutamate-induced signaling and function, activating BBB endothelial cells, killing neurons, damaging the brain, and inducing behavioral cognitive abnormalities and ataxia in animal models. Anti-NMDA-NR2A/B antibodies, in particular, cross-react with dsDNA and are correlated with neuropsychiatric, cognitive, behavioral, and mood abnormalities in SLE patients (Levite, 2014; Yang et al., 2017).

Taken together, the discovery of anti-NMDAR antibodies in NPSLE and other neurological diseases and the elucidation of their mechanisms provide potential theoretical support for the future elimination or silencing of these antibodies in certain patients through immunotherapy.

##### 2.2.4.4 Anti-aquaporin four antibodies

Aquaporin 4 (AQP4) is the major water channel protein in the central nervous system, mainly expressed at the ends of astrocytes and located at the blood-brain barrier (BBB) and the brain-cerebrospinal fluid barrier, and is responsible for controlling cellular water flow (Saikali et al., 2009). Anti-AQP4 antibodies generate astroglial damage by initiating an inflammatory immune response, activating complement-dependent cytotoxicity, leading to disruption of the BBB and causing leukocyte infiltration and cytokine release, resulting in oligodendrocyte, myelin, and neuronal damage (Rocca et al., 2020).

Neuromyelitis optica (NMO) is an immune-mediated primary inflammatory demyelinating disease of the central nervous system involving mainly the optic nerve and spinal cord, characterized by the simultaneous or short-term occurrence of optic neuritis and severe transverse myelitis. Pathogenetic serum IgG autoantibodies to aquaporin 4 (AQP4) are the primary cause of NMO in the majority of patients. AQP4 is the main target of NMO-IgG, which is considered to be an immune marker of NMO (Jarius et al., 2020). Since some patients with NMO may go on to develop SLE, and a small proportion of patients with SLE also develop demyelinating lesions that can be diagnosed as NMO, the diagnoses of NMO and SLE partially overlap (Schwartz et al., 2019). Therefore, a recent retrospective cohort study examined the relationship between SLE and anti-AQP4 antibodies and found that some juvenile SLE patients develop anti-AQP4 antibodies and are more likely to be at higher risk of neurological involvement (Moraitis et al., 2019). These suggest that it may be possible to test positive for NMO-IgG/AQP4-Ab, which perhaps could be potentially of assistance in the early diagnosis of NPSLE.

##### 2.2.4.5 Anti-endothelial antibodies (AECAs)

The antigens of anti-endothelial cell antibodies are a heterogeneous cluster of proteins located on the surface of endothelial cells that bind to vascular endothelial cells (EC) through variable region-specific interactions and destroy EC through complement-mediated or antibody-dependent cytotoxic effects, leading to vascular injury (Legendre et al., 2017). AECAs can be found in a variety of autoimmune diseases associated with vasculitis, particularly systemic vasculitis and SLE, which are hallmarks of vascular damage and vasculitis. It has been demonstrated that in patients with SLE, psychosis or depression are associated with serum anti-endothelial antibodies (Perricone et al., 2015). Nedd5 is an intracytoplasmic protein of the septin family. An endothelial cDNA library identifies the C-terminal region of Nedd5 as a novel autoantigen in SLE patients with psychiatric manifestations. Anti-Nedd5 antibodies have been discovered to be substantially associated with psychiatric symptoms in SLE (Margutti et al., 2005; Valesini et al., 2006). A recent study examined the relationship between AECAs and SLE and drew the conclusion that AECAs are involved in and mediate the initial phase of SLE vascular damage by causing EC activation and dysfunction, whereas they do not play a role in the subsequent development of vasculitis (Cieślik et al., 2022).

##### 2.2.4.6 Anti-ubiquitin carboxyl hydrolase L1 antibodies

A randomized controlled trial by Li et al. discovered that anti-UCH-L1 antibodies are a promising CSF biomarker which offers high specificity for the diagnosis of NPSLE, and elevated CSF UCH-L1 levels can reflect the clinical severity of NPSLE (Li et al., 2019). Similarly, a recent study on autoantibodies concluded that the autoantibody against amino acids 58 to 69 of UCH-L1 (UCH58-69) revealed a high degree of specificity and diagnostic relevance in distinguishing patients with NPSLE from those with SLE without neuropsychiatric symptoms. Serum anti-UCH58-69 levels are significantly higher in patients with NPSLE compared to SLE patients without neuropsychiatric symptoms and correlate with disease severity. Anti-UCH58-69 autoantibodies are likely to turn into a novel serum biomarker for the non-invasive diagnosis of NPSLE, which might be suitable for early screening and diagnosis of NPSLE (Guo et al., 2022).

##### 2.2.4.7 Anti-glyceraldehyde 3-phosphate dehydrogenas antibodies

GAPDH is recognized as a novel autoantigen associated with neuropsychiatric disorders, and research by Delunardo et al. uncovered a significant positive correlation between serum anti-GAPDH antibodies levels and harmful cognition and mood (e.g., schizophrenia and major depression) in SLE patients. Anti-GAPDH antibody levels were higher in SLE patients with psychotic symptoms than in SLE patients without psychotic symptoms (Delunardo et al., 2016). Evidence in favour of this finding is also provided by the study by Sun et al. They found that serum anti-GAPDH antibodies levels were notably elevated in patients with NPSLE and were associated with elevated SLEDAI-2K, ESR, IgG and IgM. In addition to this, it was also correlated with increased intracranial pressure and the incidence of cerebrovascular lesions. Further, the protective effect of elevated anti-GAPDH antibodies levels against seizures has the potential to make them an indicator of brain tissue damage in future clinics (Sun et al., 2019).

#### 2.2.5 Complement activation

It is well known that the complement system has a key involvement in SLE. In lupus nephritis, the relationship between renal damage and circulating immune complexes, complement deposition and anti-C1q levels is broadly recognized. Studies in recent years have proposed that complement activation is involved in the pathophysiological processes of CNS inflammation and neurodegenerative diseases and is considered to be one of the factors involved in the pathogenesis of NPSLE. A critically contributing factor to the production of circulating autoantibodies in NPSLE and the interaction with its consequential thrombotic lesions may be complement deposition dependent (Cohen et al., 2017). Previously in murine models, investigators demonstrated that inhibition of both C3aR and C5aR complement receptors by using complement inhibitors reduced neuronal apoptosis and neuronal gliosis, respectively, thereby alleviating the symptoms of NPSLE (Jacob et al., 2010a; Jacob et al., 2010b). Furthermore, Magro-Checa C et al. found correlations between diffuse NPSLE and anti-C1q, C3/AP50 as well as focal NPSLE and C4, suggesting a potential role of complement activation and complement components in cognitive dysfunction in NPSLE (Magro-Checa et al., 2016a). The efficacy of some complement receptor inhibitors in SLE is gradually being demonstrated, and the treatment of NPSLE is facilitated by complement receptor inhibition in a mouse model, and further exploration of the connection may allow us to find a breakthrough in the treatment of NPSLE in terms of complement system and complement deposition.

#### 2.2.6 Inflammatory mediators

Inflammatory mediators can cause neurological diseases by disrupting the BBB. Neuronal or glial cells may also produce these cytokines and chemokines within the intrathecal, which might increase the permeability of the BBB, thus facilitating the entry of circulating autoantibodies and leukocytes into the CNS.

Inflammatory cytokines such as tumor necrosis factor-α (TNF-α), TNF-like weak inducer of apoptosis (TWEAK), interferon-γ (IFN-γ), interleukin-6 (IL-6), interleukin-8 (IL-8), and B-cell activating factor (BAFF) have been detected in the cerebrospinal NPSLE patients, suggesting a key role of inflammatory response in the development of NPSLE (Kivity et al., 2015). Yoshio et al. observed that intrathecal concentrations of IL-6, IL-8, monocyte chemotactic protein 1 (MCP-1)/CCL2, IFN-γ inducible protein-10 (IP-10)/CXCL10, granulocyte colony-stimulating factor (G-CSF) and granulocyte-macrophage CSF (GM-CSF) were not affected by serum concentrations in patients with central NPSLE indicating that the production of chemokines by these cytokines probably occurs in the CNS and may be a prerequisite in the pathogenesis of central NPSLE (Yoshio et al., 2016).

There is robust previous evidence that IFN-α can cause neuropsychiatric manifestations in human and mouse animal models, and Santer et al. suggest that serum and/or CSF from NPSLE patients may contain unusually high activity for IFN-α induction (Santer et al., 2009). Besides, blocking IFN-α signaling reduces microglia-associated synaptic loss and alleviates anxiety-like behavior and cognitive deficits in 564Igi lupus-prone mice (Bialas et al., 2020).

Type III interferon (IFN-λ) is the most recently identified cytokine in the interferon family. Several recent interferon-related studies have discovered elevated serum concentrations of IFN-λ in several autoimmune diseases, including SLE, implying that IFN-λ may be involved in the regulation of cellular and humoral immunity in SLE. In a pristane-induced lupus model, mice with defective IFN-λ receptors survived at a higher rate in the early stages of autoimmune development, had a reduced rate of lipid granuloma formation compared to pristane-treated wild-type mice, and a decrease in anti-dsDNA autoantibody titres was also observed. This mechanism may be mediated through the activation of keratin-forming cells and thylakoid cells, thereby recruiting immune cells and producing chemokines that promote tissue inflammation involved in the induction of immune dysregulation and tissue inflammation (Goel et al., 2020; Aschman et al., 2021).

Of the reported cytokines, IL-6 is deemed to have the strongest positive correlation with NPSLE, with a significant increase in IL-6 expression throughout the CNS in NPSLE patients. In addition, there is evidence from various studies that IL-6 in the CSF is likely to be a valid marker for the diagnosis of central NPSLE, with high sensitivity and specificity. In a further specific classification of NPSLE in the latest research, serum IL-6 and CSF IL-6 were found to be significantly elevated in acute confusion states (ACS) compared to non-ACS diffuse NPSLE (anxiety disorders, cognitive dysfunction, mood disorders and psychosis) or focal NPSLE. Additionally, Q albumin (CSF/serum albumin quotient) was also substantially higher in ACS than in the other two groups of NPSLE, but notably Q albumin appeared to be more associated with serum IL-6 than CSF IL-6 in patients with diffuse NPSLE (both ACS and non-ACS) (Hirohata & Kikuchi, 2021).

In terms of brain intrinsic immunity, a recent study found that microglia hyperactivation exists in the brain of SLE model (MRL/lpr) mice. The engagement of hippocampal microglia by CD40 was involved in the development of NPSLE cognitive dysfunction and blocking microglia activation can reduce neuropsychiatric symptoms in mice (Qiao et al., 2021).

Some recent studies have focused on salivary inflammatory markers and they have found that salivary cytokines may be potential diagnostic biomarkers for SLE, where salivary IL-6 can be used to reliably assess the inflammatory process of SLE (Stanescu et al., 2018; Zian et al., 2021).

Each of these cytokines is more or less involved in the pathogenesis of NPSLE and may be promising targets for the treatment of the neuropsychiatric symptoms and systemic manifestations of SLE. Nevertheless, not all cytokines are appropriate for detection in serum or CSF for NPSLE, and there is currently a lack of clarity regarding their specific identification targets. Nevertheless, emerging and meaningful biomarkers for the diagnosis of NPSLE are being explored and demonstrated consistently. With the exception of IL-6, which has been shown to have the strongest positive correlation with NPSLE as mentioned earlier, and IL-10, which is relevant to disease activity (Jeltsch-David & Muller, 2014), several other autoantibodies and proteins, such as anti-microtubule-associated protein two antibodies in cerebrospinal fluid and osteopontin, are gradually being considered as biomarkers for the diagnosis of NPSLE (Yamada et al., 2016; Kitagori et al., 2019). It seems that in clinical work it may be possible to detect and diagnose NPSLE as early as possible and achieve secondary prevention of the disease by preferential detection of these biomarkers. The development of new safe and effective methods for the detection of various cytokines is called for in the future and may hold promise for the clarification of the pathogenesis of NPSLE.

### 2.3 Management and treatment

The diversity of neuropsychiatric symptoms in patients with NPSLE and the limited understanding of its complex pathogenesis have limited the development of targeted therapies. In 2010, the European League Against Rheumatism (EULAR) issued a consensus recommendation for the management of NPSLE. These recommendations state that neuropsychiatric manifestations in patients with SLE should first be assessed and treated in the same way as in patients without SLE, including routine symptomatic therapy, psychological intervention, secondary prevention of some corresponding complications, and alleviation of the underlying causes and exacerbating factors, before attributing them to SLE and managing them as appropriate (G. K. Bertsias et al., 2010).

Current treatments are primarily symptomatic and include the use of antipsychotic and antidepressant medications and anti-anxiety medications to treat manifest psychiatric symptoms, antiepileptic drugs to treat seizures, or immunosuppressive agents (e.g., corticosteroids, cyclophosphamide, azathioprine, mycophenolate mofetil) to suppress systemic inflammatory responses. Of these, high-dose glucocorticoids and intravenous cyclophosphamide remain the most fundamental treatments for patients with severe symptoms, which also reflect the systemic inflammatory response as well as the potential autoimmune process. In particular, azathioprine and mycophenolate have also been used for the maintenance treatment of SLE and for mild to moderate neuropsychiatric symptoms. Additionally, rituximab, intravenous immunoglobulin or plasma exchange are alternative therapies that may be considered in the absence of a response to these drugs (Magro-Checa et al., 2016b).

In the last decade or so, targeted therapy with emerging biological agents has also emerged as a new treatment option. Although several biological agents have been tried for the treatment of SLE with some clinical efficacy, only belimumab has been approved by the U.S. Food and Drug Administration for the treatment of SLE. Unfortunately, however, NPSLE is not an approved indication for belimumab and it has not been assessed in the management of central nervous system symptoms. Notwithstanding that, a retrospective observational cohort study by Plüß M et al. found a favorable effect of belimumab on neuropsychiatric symptoms in patients with severe NPSLE (Plüß et al., 2020). Rituximab is a chimeric monoclonal antibody with a human IgG1 structural domain and a murine CD20 variable region that binds specifically to the B-cell marker CD20 antigen on the cell membrane and belongs to a classical B-cell clearance therapy. Previous related studies have reported that rituximab treatment can rapidly improve CNS-related manifestations of NPSLE patients, including the state of acute confusion, cognitive dysfunction and seizures, and even reduce the expression of CD40L and CD69 (Tokunaga et al., 2007). With the exception of NPSLE, the beneficial effects of rituximab in other inflammation-related neurological disorders are gradually being identified and confirmed, which may indicate that we can find their underlying relevance in these diseases (Whittam et al., 2019). The effectiveness of additional types of biological agents for symptom relief in NPSLE is still uncertain, which is an area where future research regarding neuropsychiatric symptoms in SLE could be pursued.

Apart from the immunosuppressants mentioned above, treatment of thrombotic or embolic manifestations of NPSLE, such as antiphospholipid antibody syndrome (APS), requires the use of anticoagulation or antiplatelet therapy. Patients with APS must be prevented from developing systemic recurrent thrombosis and usually require lifelong anticoagulation. Antiplatelet agents, antimalarials and statins are all necessary to prevent recurrent thrombosis in patients (Magro-Checa, Zirkzee, et al., 2016). Recently, cationic nanomaterials have been considered for the inhibition of inflammatory responses in autoimmune diseases. Although the current application is still in its infancy and relevant studies are comparatively limited, it has shown potential in murine models of SLE. We may also look forward to its upcoming use in the treatment of NPSLE (Xie et al., 2021).

## 3 Conclusion

On the whole, NPSLE is a disease with a complex pathogenesis and is also one of the major causes of death in patients with SLE. Although in recent years an increasing number of biomarkers and autoantibodies have been tested and advanced imaging techniques have provided effective tools to clarify the diagnosis of NPSLE. Unfortunately, there is still no ‘gold standard’ for the diagnosis of NPSLE. Due to the diversity and rarity of its neuropsychiatric manifestations and the complexity of its pathophysiology, it seems unlikely that a uniform standard can be established for the diagnosis of NPSLE. The diagnosis currently relies on a detailed clinical assessment of the patient and appropriate examinations of serology and imaging, and neuropsychiatric symptoms can be attributed to NPSLE if other disorders are ruled out. Similarly, due to the lack of evidence from high-quality clinical treatment trials for NPSLE, treatment is currently based on empirical symptomatic therapy. There is still a large number of unanswered questions regarding NPSLE. The existing recognized pathogenesis and the autoantibodies and biomarkers thought to be potentially associated with NPSLE are still only the tip of the iceberg. We expect future research to identify reliable biomarkers associated with neuropsychiatric symptoms of SLE or to develop and implement advanced neuroimaging examinations as well as laboratory assessments to identify early pathophysiologic changes in NPSLE to inform diagnosis and treatment. In the future, we hope that the pathogenesis and pathophysiology of NPSLE will be further clarified to guide treatment decisions as well as targeted therapies, and we look forward to seeing the light of day for NPSLE soon, thereby increasing survival rates and improving patient prognosis.
